# Reliability enhancement of EM-based tuning of microwave components using regularized operating band scanning

**DOI:** 10.1038/s41598-025-13107-y

**Published:** 2025-07-31

**Authors:** Slawomir Koziel, Anna Pietrenko-Dabrowska

**Affiliations:** 1 Engineering Optimization & Modeling Center, Reykjavik University, 101 Reykjavík, Iceland; 2 Faculty of Electronics, Telecommunications and Informatics, Gdansk University of Technology, 80-233 Gdańsk, Poland

**Keywords:** Microwave circuits, Design automation, EM-based optimization, Target band scanning, Regularization, Electrical and electronic engineering, Computational science

## Abstract

Numerical optimization is ubiquitous in contemporary microwave passive component design. Reliable quantification of electromagnetic (EM) cross-coupling, substrate losses, or environmental effects (e.g., connectors, housing) is impossible using analytical or equivalent network models. Consequently, the designs rendered using such tools must be further tuned at the EM simulation level. Although local algorithms are predominantly employed for this purpose, they are likely to fail if the starting point is of insufficient quality. On the other hand, global search techniques are associated with tremendous computational expenses. This study suggests an innovative technique for immunizing local algorithms against poor starting points, enabling their quasi-global search capability. The presented methodology reformulates the underlying optimization task by evaluating the circuit performance concerning the target operating bands scanned within the predefined ranges and by adding penalty factors proportional to the distance between the original and the scanned targets. The penalized objective is computed as the minimum of the above combination over the entire scanning range. As a result, the design specifications become attainable through local optimization over considerably broader frequency spectra at the expense of only a slight increase in the algorithm running costs. The presented approach is comprehensively verified using several microstrip components and the sensitivity-based routine as the search algorithm. The results unanimously showcase the ability of the procedure to allocate the optimum even under troublesome scenarios (poor initial points), which are unmanageable by conventional algorithms.

## Introduction

The development of contemporary microwave circuits faces numerous challenges, in large part related to performance demands induced by various application areas (mobile communications, wearable electronics, the internet of things, energy harvesting, RFID, and virtual reality equipment^1–6^. These include specific functionalities the circuits are supposed to realize (broad-band and multi-band operation, tunability, harmonic suppression^7–10^, and—more and more often—a small physical size^11–15^. Miniaturization is particularly troublesome for passive components due to physical restrictions associated with the guided wavelength^16^. It requires the incorporation of dedicated techniques (transmission line folding^17^, utilization of slow-wave phenomenon^18^, defected ground structures^19^ or metamaterials^20^. Fulfilling stringent requirements leads to geometrically sophisticated structures, the design of which is heavily reliant on electromagnetic (EM) simulation tools as well as numerical optimization procedures necessary to enhance the circuit’s performance in a reliable manner^21,22^.

Leaving alone informal approaches (e.g., variations of parameter sweeping aided by engineering experience), rigorous parameter tuning of microwave components is primarily carried out using local optimization algorithms^23^, often variations of gradient-based routines^24^, but also derivative-free techniques (generic pattern search^25^, a Hooke-Jeeves algorithm^26^, a Nelder-Mead algorithm^27^, etc.). Their computational complexity is typically acceptable from a practical perspective, whereas the procedures are mature and supported by theoretical results^28^. Furthermore, they are reliable, e.g., convergent under mild assumptions regarding the analytical properties of the underlying functions^29^. Notwithstanding, due to their inherently local operation, the said algorithms are prone to failure if the starting point (e.g., obtained with the equivalent network model) is outside the attainability region of the optimum^30^. This problem is aggravated for multi-band circuits or when redesigning the structure regarding broad ranges of center frequencies, where the likelihood of at least one of the center frequencies being severely misaligned with the target increases dramatically^31^.

In principle, the difficulties associated with the reliability of local search procedures may be mitigated by defaulting on global optimization techniques^32–35^. Yet, this possibility is employed sparsely due to the associated computational costs. For at least two decades, global optimization was predominantly realized using bio-inspired population-based routines^36,37^. These methods are tremendously expensive: the typical costs range from a few to many thousands of merit function calls. For EM-driven design, such expenses are simply unmanageable. Global optimization may be expedited by incorporating surrogate modeling methods^38–41^, usually within machine learning (ML) frameworks^42,43^, where the behavioral model is used as a fast predictor (to generate infill points presumably approaching the optimum) and iteratively improved by exploring the accumulated EM simulation data^44^. The shortcoming of these approaches is building the surrogate model. It is seriously impeded by dimensionality issues, the need to represent highly nonlinear microwave circuit responses, and typically wide ranges of geometry parameters. Consequently, ML-based algorithms are often illustrated using low-dimensionality test cases^45,46^. When applied to more challenging problems, identifying a global optimum becomes questionable. Moving back to local techniques, in^47^, a regularization approach was suggested, where the objective function is augmented by the term proportional to the discrepancy between the actual and intended center frequencies. This formulation allows for smoothening the merit function, enhancing the local search process’s immunity to poor initial designs. However, these benefits are associated with increased algorithmic complexity and limited generality as the circuit operating frequencies must be determined based on EM-simulated characteristics through appropriate pre-processing^47^.

This study suggests a new technique for enhancing the reliability of microwave optimization. It effectively enables a quasi-global search capability by means of localized algorithms. The foundation of the presented approach is to reformulate the design task by assessing the circuit performance concerning the target operating bandwidth (or bandwidths for multi-band structures) scanned within the pre-determined frequency ranges. The objective function is complemented by adding a regularization term, which monotonically increases as a function of the distance between the original and scanned targets. Finally, the penalized objective is defined as the minimum of the scanned (and regularized) objective over the assumed frequency scanning spectra. The outlined approach alters the characteristics of the optimization problem in two ways: (i) dramatically expands the frequency range over which the circuit’s operating parameters fall into the optimum’s region of attraction, and (ii) enforces relocation of the circuit center frequencies towards their intended values. Consequently, local algorithms are immunized against poor initial designs. From a design utility point of view, the aforementioned design specification scanning enables globalized search without incurring computational costs customarily associated with widespread parameter space exploration. Our methodology was extensively showcased with the help of several microstrip components and the trust-region procedure employed as the core search procedure. The results conclusively corroborate the efficacy of the discussed method regarding reliability, superiority over conventional formulation, and computational efficiency.

## Enhanced-reliability microwave optimization by target frequency scanning

In this section, we introduce the proposed target scanning technique, the associated reformulation of the EM-based design task, and the exemplary search procedure to be used for illustration and verification purposes in Sect. 3.

The material arrangement is the following. Section 2.1 recalls a conventional statement of the EM-based microwave design problem. Section 2.2 discusses the target frequency scanning concept and the resulting penalized objective function. The completed optimization procedure is discussed in Sect. 2.3.

### Microwave design optimization. Formulation of the problem

We start our consideration by stating the EM-based design optimization problem. Adjustable parameters of the circuit at hand will be incorporated into ***x*** = [*x*_1_ … *x*_*n*_]^*T*^. For passive structures, these are typically variables controlling the circuit geometry (length and widths of metallization elements, gaps between transmission lines or patches, etc.). The aggregated circuit responses will be denoted as ***R***(***x***), which are typically scattering parameters versus frequency *S*_*kj*_(***x***,*f*) (here, *k* and *j* are port indices, whereas *f* stands for frequency), or quantities derived therefrom (e.g., the phase response). The design merit is evaluated with an objective function *U*. In this work, *U* is assumed to depend on ***x*** and the target operating bands, marked as *F*. For multi-band circuits, we would have *F* = *F*_1_ ∪ … ∪ *F*_*K*_, with *F*_*k*_ = [*f*_*k*.1_ *f*_*k*.2_]. Here, *f*_*k*.1_ and *f*_*k*.2_ denote the edges of the *k*th frequency range of interest, whereas *K* is the overall number of bands.

Function *U* is defined as monotonically increasing regarding design objective. Several examples of the merit function formulation have been included in Table 1. Given the aforementioned properties, the EM-based design endeavor is posed as

1 $${\mathbf{x}^*} = \arg \;\mathop {\min }\limits_{\mathbf{x} \in X} U\left( {\mathbf{x},F} \right)$$

The optimization process is conducted over the space *X*, which is customarily decided upon based on the circuit parameter bounds. Possible extra constraints may be handled directly^48^ or indirectly, using penalty functions^49^. The latter is more convenient to handle computationally intensive conditions (i.e., evaluation of which necessitate EM simulation), as indicated in Table 1.

**Table 1:** EM-driven design tasks. A description of the problem (left) is supplemented by a possible analytical formulation of the merit function (right).

Design task	Objective function*
Circuit: Impedance matching transformer Objectives: Minimize maximum in-band reflection Target operating band: *F* = [*f*_1_ *f*_2_]	$U({\boldsymbol{x}})=\hbox{max} \{ f \in [{f_1}\;{f_2}]:|{S_{11}}({\boldsymbol{x}},f)|\}$
Circuit: Microstrip coupler Objectives: Minimize matching and isolation at the target operating frequency *f*_0_ Ensure the required power split *K*_*P*_ at *f*_0_ Target operating band: *F* = {*f*_0_}	$$\begin{gathered} U({\boldsymbol{x}})=\hbox{max} \left\{ {|{S_{11}}({\boldsymbol{x}},{f_0})|,|{S_{41}}({\boldsymbol{x}},{f_0})|} \right\}+ \\ +\beta {\left[ {|{S_{21}}({\boldsymbol{x}},{f_0})| - |{S_{31}}({\boldsymbol{x}},{f_0})| - {K_P}} \right]^2} \\ \end{gathered}$$
Circuit: Dual-band coupler Objectives: Minimize matching and isolation at the operating frequencies *f*_1_ and *f*_2_ Ensure equal power split at *f*_1_ and *f*_2_ Target operating band: *F* = {*f*_1_} ∪ {*f*_2_}	$$\begin{gathered} U({\boldsymbol{x}})=\hbox{max} \{ |{S_{11}}({\boldsymbol{x}},{f_1})|,|{S_{11}}({\boldsymbol{x}},{f_2})|,|{S_{41}}({\boldsymbol{x}},{f_1})|,|{S_{41}}({\boldsymbol{x}},{f_1})|\} \\ +\beta \left[ {{{(|{S_{21}}({\boldsymbol{x}},{f_1})| - |{S_{31}}({\boldsymbol{x}},{f_1})|)}^2}+{{(|{S_{21}}({\boldsymbol{x}},{f_2})| - |{S_{31}}({\boldsymbol{x}},{f_2})|)}^2}} \right] \\ \end{gathered}$$
Circuit: Compact rat-race coupler Objectives: Minimize the footprint area *A*(***x***) Ensure equal power split at the operating frequency *f*_0_ Maintain |*S*_11_|, |*S*_41_| ≤ − 20 dB over the bandwidth 2*B* centered at *f*_0_ Target operating band: *F* = [*f*_0_ – *B*, *f*_0_ + *B*]	$$\begin{gathered} U({\boldsymbol{x}})=A({\boldsymbol{x}})+{\beta _1}{\left[ {\hbox{max} \{ c({\boldsymbol{x}})+20,0\} /20} \right]^2} \\ +{\beta _2}{\left[ {|{S_{21}}({\boldsymbol{x}},{f_0})| - |{S_{31}}({\boldsymbol{x}},{f_0})|} \right]^2} \\ \end{gathered}$$ where$$\begin{gathered} c({\boldsymbol{x}})=\hbox{max} \{ f \in [{f_0} - B,{f_0}+B]: \\ \hbox{max} \{ |{S_{11}}({\boldsymbol{x}},f)|,|{S_{41}}({\boldsymbol{x}},f)|\} \\ \end{gathered}$$
Circuit: Dual-band coupler Objectives: Implement the circuit on the substrate of relative permittivity *ε*_*r*_ Minimizes |*S*_11_| and |*S*_41_| at the operating frequencies *f*_1_ and *f*_2_ Ensure equal power split at *f*_1_ and *f*_2_ Target operating band: *F* = {*f*_1_} ∪ {*f*_2_}	$$\begin{gathered} U({\boldsymbol{x}})=\hbox{max} \left\{ {|{S_{11}}({\boldsymbol{x}},{f_1})|,|{S_{41}}({\boldsymbol{x}},{f_1})|,|{S_{11}}({\boldsymbol{x}},{f_2})|,|{S_{41}}({\boldsymbol{x}},{f_2})|} \right\} \\ +\beta {\left[ {|{S_{21}}({\boldsymbol{x}},{f_1})| - |{S_{31}}({\boldsymbol{x}},{f_1})|} \right]^2}+\beta \left[ {|{S_{21}}({\boldsymbol{x}},{f_2})| - |{S_{31}}({\boldsymbol{x}},{f_2})|} \right] \\ \end{gathered}$$
Circuit: Triple-band power divider Objectives: Ensure equal power split at the operating frequencies *f*_1_, *f*_2_, and *f*_3_ Minimize input matching |*S*_11_|, output matching |*S*_22_| and |*S*_33_|, and isolation |*S*_23_| at *f*_1_, *f*_2_, and *f*_3_ Target operating band: *F* = {*f*_1_} ∪ {*f*_2_} ∪ {*f*_3_}	$$\begin{gathered} U({\boldsymbol{x}})=\hbox{max} \{ \mathop {\hbox{max} }\limits_{{k,l \in \{ 1,2,3\} }} |{S_{kk}}({\boldsymbol{x}},{f_l})|,\mathop {\hbox{max} }\limits_{{l \in \{ 1,2,3\} }} |{S_{23}}({\boldsymbol{x}},{f_l})|\} \\ +\beta \sum\limits_{{l=1}}^{3} {{{(|{S_{21}}({\boldsymbol{x}},{f_l})| - |{S_{31}}({\boldsymbol{x}},{f_l})|)}^2}} \\ \end{gathered}$$

*The terms followed by *β* are penalty factors introduced to enforce the design constraints (e.g., required power split or matching/isolation bandwidth).

### Target frequency scanning. The concept and analytical formulation

**Fig. 1:**
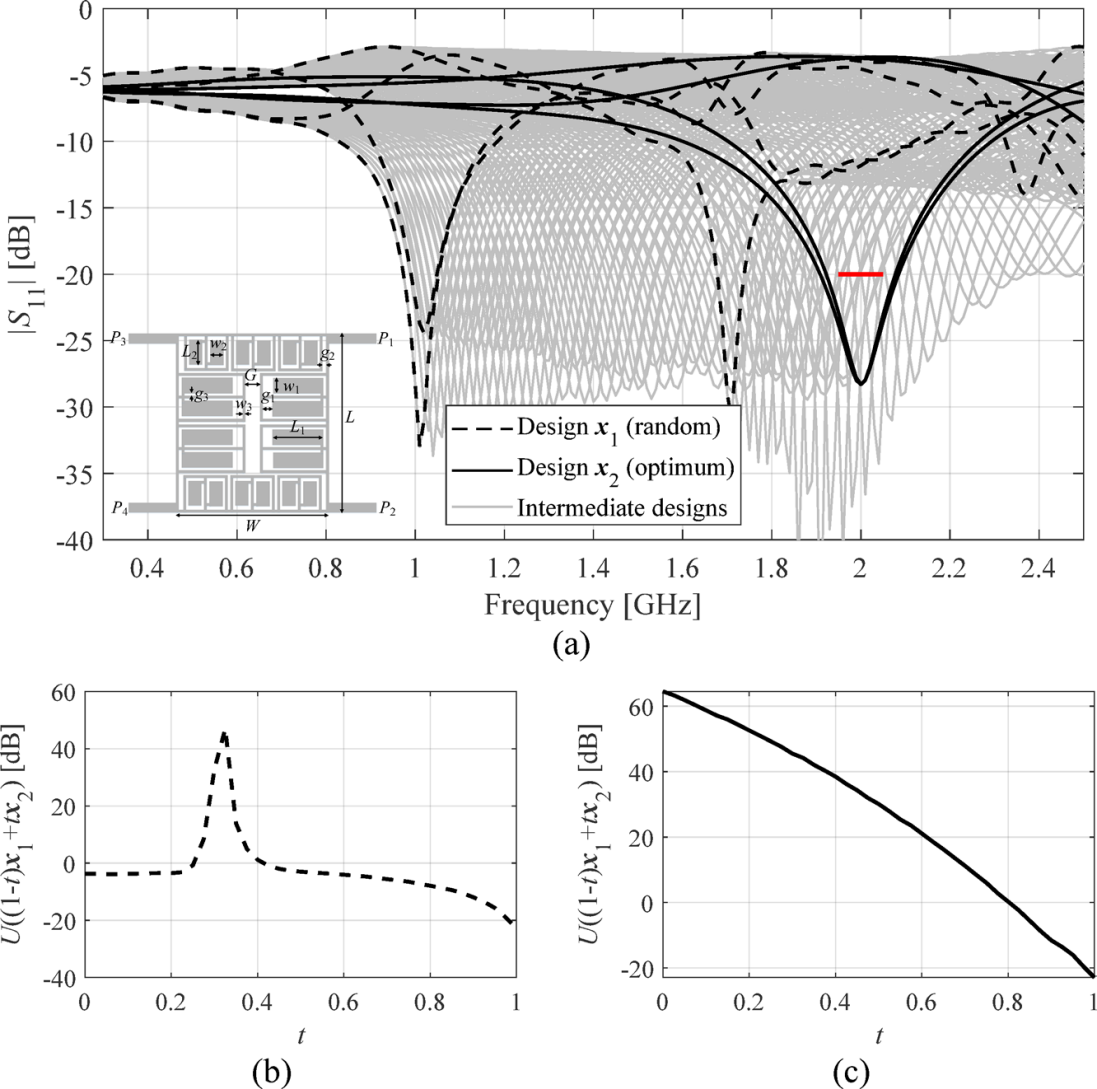
Compact branch-line coupler: (**a**) *S*-parameters at two designs: ***x***_1_ (random), and ***x***_2_ – optimum (best matching) design w.r.t. *F* = [1.95 2.05] GHz (marked red). Circuit geometry shown in the inset. Grey plots correspond to intermediate designs ***x***(*t*) = (1–*t*)***x***_1_ + *t****x***_2_, 0 ≤ *t* ≤ 1; (**b**) the merit function *U* along the segment ***x***(*t*): solving (1) with *U*(***x***) = max{*f* ∈ *F* : |*S*_11_(***x***,*f*)|,|*S*_41_(***x***,*f*)|} + *β*[|*S*_21_(***x***,*f*_0_)| – |*S*_31_(***x***,*f*_0_)|] (minimax optimization of matching and reflection regarding *F*, and the enforcement of equal power division at *f*_0_ = 2 GHz) will fail when using local algorithm as the merit function *U* has a maximum between ***x***_1_ and ***x***_2_; (**c**) the penalized merit function *U*_*P*_ of (3) along ***x***(*t*): as the objective function is monotonically decreasing when moving from design ***x***_1_ to ***x***_2_, the optimum is now reachable through local search when initiated from ***x***_1_.

Our objective is to enhance the reliability of local search by reducing the likelihood of obtaining inferior-quality design due to poor starting points. The problem has been illustrated in Fig. 1. We aim to improve the coupler reflection coefficient and isolation over the frequency band 1.95 GHz to 2.05 GHz and maintain equal power division at *f*_0_ = 2 GHz. The optimum design ***x***_2_, is unreachable from the design ***x***_1_ when using local optimization techniques and the objective function *U*, because ***x***_1_ and ***x***_2_ are separated by a local maximum of *U* (cf. Figure 1b), impassable by methods such as gradient-based search.

We reformulate the design task so local optimization does not fail under the aforementioned circumstances. The main prerequisites are as follows:

- The target operating bands *F*_*k*_, *k* = 1, …, *K*, are scanned within the pre-defined range *D*_*F*_ = [–*D*_*f*_, *D*_*f*_];
- The original objective function *U* is computed for each shifted band *F*_*k*_ + *d*_*f*_, *d*_*f*_ ∈ *D*_*F*_ (in practice, it is realized for a discrete set of shifts with in *D*_*F*_);
- A penalty factor *P*(*d*_*f*_) is evaluated and added to the re-calculated objective function.

The term *P*(*d*_*f*_) is defined to monotonically increase as a function of |*d*_*f*_| to favor the designs for which the actual operating frequency is closer to the target

2 $$P({d_f})=M{\left( {\frac{{|{d_f}|}}{{{D_f}}}} \right)^\alpha }$$

where *M* is the maximum penalty (here, set to 10^2^); *α* > 0 is the shape parameter (here, set to unity).

Scanning the target bandwidths permits identifying the operating frequency location and assigning low values of the original objective function therein. Meanwhile, the penalty term *P* allows for enforcing relocation of the operating frequencies toward the targets.

The penalized objective function *U*_*P*_ is evaluated by computing the original function *U* for all combinations of operating band shifts, and by augmenting it with the penalty terms. Thus, for a *K*-band circuit, we define

3 $$\begin{gathered} {U_P}({\boldsymbol{x}},F)={U_P}({\boldsymbol{x}},{F_1} \cup ... \cup {F_K})= \\ =\mathop {\hbox{min} }\limits_{{{d_{f1}},...,{d_{fK}} \in {D_F}}} \left\{ {U({\boldsymbol{x}},{F_1}+{d_{f1}} \cup ... \cup {F_K}+{d_{fK}})+\sum\nolimits_{{k=1}}^{K} {P({d_{fk}})} } \right\} \\ \end{gathered}$$

The original design task (1) can now be reformulated as a penalized one using *U*_*P*_

4 $${{\boldsymbol{x}}^*}=\arg \mathop {\hbox{min} }\limits_{{{\boldsymbol{x}} \in X}} {U_P}({\boldsymbol{x}},F)$$

The benefit of this formulation is that even for center frequencies remote from the targets yet within the scanning range *D*_*F*_, they can be identified by assigning low values of *U*(***x***,*F*_1_ + *d*_*f*1_ ∪ … ∪ *F*_*K*_ + *d*_*fK*_) for a combination of shifts corresponding to the actual allocation of the center frequencies. The penalty terms implement the monotonicity of *U*_*P*_ regarding the differences between the intended and actual center frequencies, thereby enforcing the former to migrate towards the targets (leading to a reduction of *P*(*d*_*fk*_)).

**Fig. 2:**
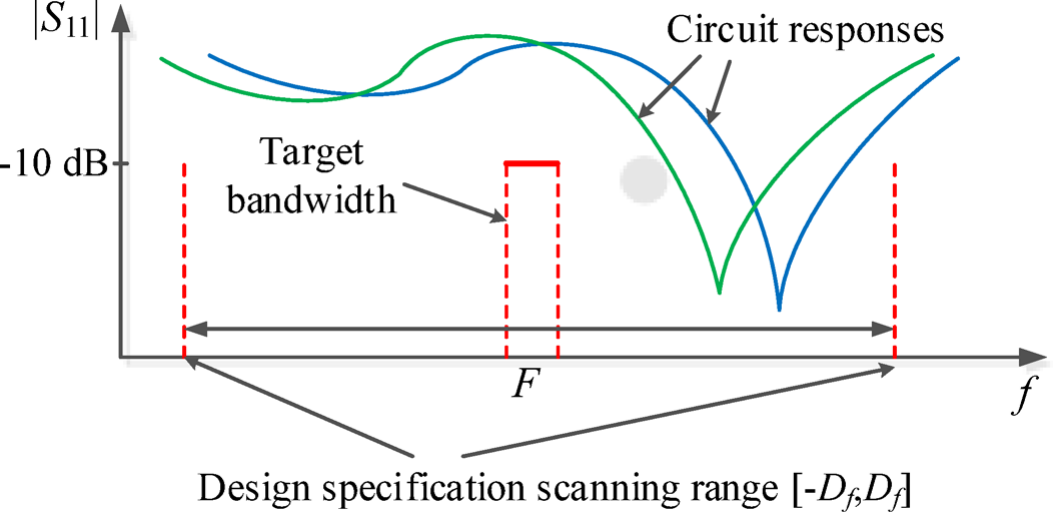
The concept of penalized optimization: (**a**) exemplary target bandwidth *F*, the specification scanning range *D*_*f*_, and two exemplary responses (marked as 1 and 2); (**b**) two exemplary instances of shifting the target bandwidth (by *d*_*f*1_ and *d*_*f*2_). The original objective function *U* is evaluated for shifted *F*, and a penalty factor is added, which is monotonically dependent on the shift |*d*_*f*_|, cf. (2); (**c**) penalized objective *U*_*P*_ is computed as the minimum of *U* with added penalty factors computed over the entire scanning range [−*D*_*f*_, *D*_*f*_] (shown as circles for exemplary shifts *d*_*f*1_ and *d*_*f*2_). Penalized objective value is lower for response 1 than for response 2, which indicates that design relocations towards the original target are promoted.

Figure 2 graphically explains the penalized objective function concept. Suppose the circuit’s center frequency does not differ from the target bandwidth more than *D*_*f*_. Then the scanning procedure allows identifying its location and assign an appropriate value to *U*_*P*_. The latter combines the actual performance concerning the original merit function *U* concerning the shifted target bandwidth, and a penalty term *P*. In practice, the scanning range *D*_*f*_ is set relatively large to accommodate even relatively poor starting points. The maximum penalty level *M* is set at high values (here, 10^2^) to promote reducing the differences between the assumed and the actual center frequency (see Fig. 2 (panels (b) and (c)).

For the coupler circuit of Fig. 1(a), the penalized objective does resolve the issue demonstrated in Fig. 1(b). According to Fig. 1(c), the function *U*_*P*_(***x***) monotonically decreases towards the optimum when computed over the segment ***x***(*t*) = (1–*t*)***x***_1_ + *t****x***_2_, 0 ≤ *t* ≤ 1. The latter ensures that the optimum ***x***_2_ can be reached from ***x***_1_ by means of local search.

It should also be noted that the proposed approach (formulation (2), (3)) and the original problem (1) are equivalent in the following sense. If the optimum design is attainable, the parameter vector minimizing (3) coincides with the solution to (1). This is because the penalty terms *P*(*d*_*f*_) of (2) becomes zero for *d*_*f*_ = 0, which implies that *U*_*P*_(***x***,*F*) → *U*(***x***,*F*).

It is also worth noting that our method is best suited for problems in which the underlying objective function exhibits sharp local minima (e.g., frequency allocation of the resonances). This is because it enables locating such minima from distant starting points, which is beyond the reach of the conventional approaches. Furthermore, the specific type of response being optimized (e.g., *S*-parameters) is not relevant.

### Optimization algorithm

**Fig. 3:**
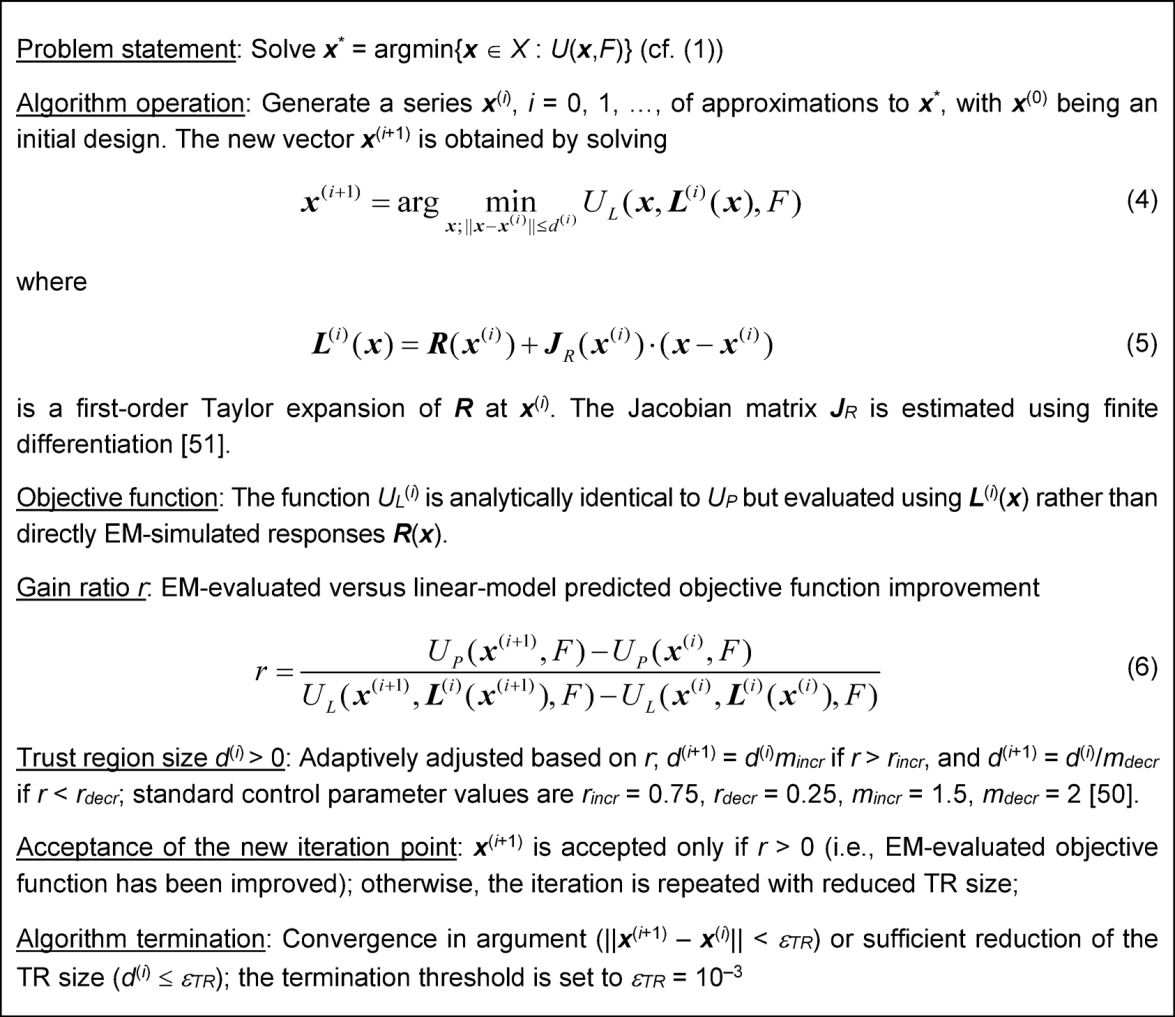
Operating principles of the standard TR procedure.

The penalized optimization approach can be coupled with diverse local optimization procedures, as the implemented mechanisms are pertinent to the problem formulation and encoded in the objective function. In particular, our method may be considered as an overlay on the objective function and be applied in conjunction with gradient-based optimization algorithms. However, the specific procedure employed must be iterative in nature, i.e., in each iteration, a certain step toward a minimum is taken whose size is governed by the objective function improvements (or by our overlay in the case of the proposed technique). Here, for demonstration, the proposed mechanism is embedded into the trust-region (TR) algorithm^50^ using finite differentiation (FD) to evaluate the circuit’s response gradients^51^. The TR routine renders approximated solutions ***x***^(*i*)^, *i* = 0, 1, 2, …, presumably approaching x^*^. Subsequent parameter vectors are produced by optimizing the circuit outputs’ first-order Taylor expansion model. The procedure’s outline is summarized in Fig. 3.

It should be emphasized that the essence of the proposed methodology is a penalized reformulation of the objective function, which regularizes the optimization task, facilitating optimum identification. This does not affect the computational complexity of the underlying search algorithm, which, in this work, is the trust-region gradient-based routine. The computational complexity of this algorithm scales linearly with the problem dimensionality (per iteration), with the main cost contributor being estimation of the circuit response Jacobians using finite differentiation.

A comment should be made concerning the choice of the TR algorithm as the underlying search routine. TR is a convenient approach in terms of constraint handling. Each candidate design produced by the algorithm is obtained by optimizing the linear approximation model of the circuit responses. Consequently, any constrained optimization procedure can be applied (the linear model is cheap to evaluate, and EM analysis is only performed to verify the new design). In this study, we use the sequential quadratic programming (SQP) algorithm implemented in the MATLAB Optimization Toolbox^52^.

## Results

Here, we demonstrate the penalized optimization approach elucidated in Sect. 2 with the help of several microwave devices, including two coupling circuits and a power divider. For all test problems, the search process starts from several initial designs, assigned to be intentionally troublesome of conventional local search. The latter is verified by carrying out local search according to the standard formulation (1) (cf. Section 2.1). The primary question is whether design specification scanning and the formulation (2), (3) are capable of immunizing local (here, gradient-based) optimization process against poor initial conditions.

The material is organized into three subsections. Section 3.1 introduces the considered verification cases. The setup and numerical outcomes are encapsulated in Sect. 3.2. Section 3.3 puts together the findings and evaluates the performance of the proposed technique.

**Fig. 4:**
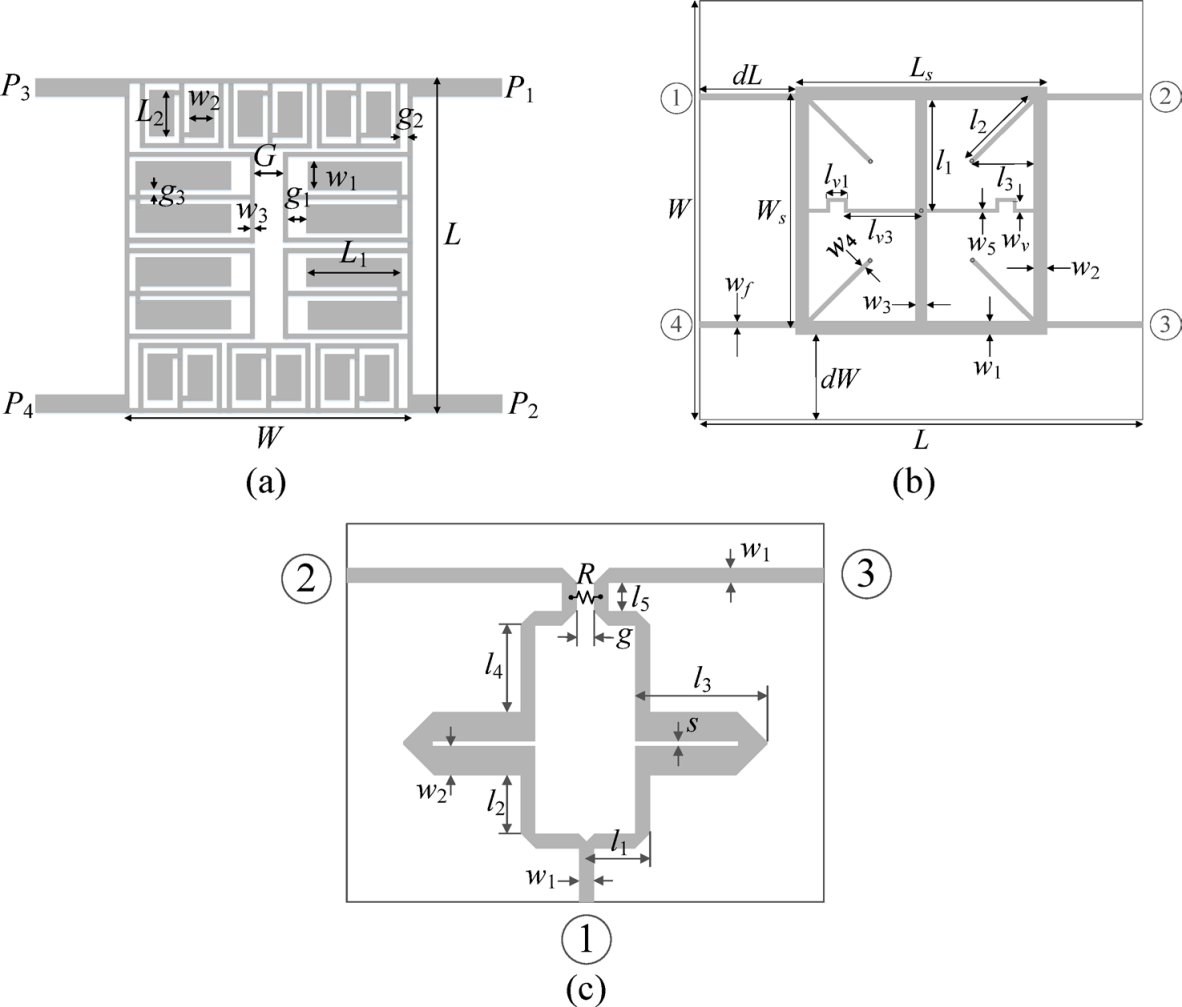
Test cases: (**a**) Circuit I^53^, (**b**) Circuit II^54^, (**c**) Circuit III^55^, *R* indicates a lumped component (100-ohm resistor).

**Table 2:** Verification case I: critical data and design objectives.

Parameter	Description
Substrate	FR4 (*ε*_*r*_ = 4.4, *h* = 1.0 mm)
Design parameters	***x*** = [*G g*_1_ *g*_2_ *g*_3_ *w*_1_ *w*_3_ *L*_1_ *L*_2_]^*T*^
Other parameters	*L* = 4*w*_1_ + 10*w*_3_ + 15*g*_3_ + 2*L*_2_, *W* = 4*w*_3_ + 2*L*_1_ + *G* + 2*g*_1_ + 2*g*_3_
EM model	CST Microwave Studio
Target operating frequencies [GHz]	*F*_1_ = [1.95 2.05] GHz
Design goals	Minimize matching |*S*_11_| and isolation |*S*_41_| at the operating frequency band *F*_1_ Ensure power split ratio *K*_*P*_ = 0 dB at the operating frequency *f*_0_ = 2.0 GHz
Parameter space *X*	***l*** = [0.2 0.2 0.2 0.2 1.0 0.2 4.0 2.0]^*T*^ ***u*** = [1.5 1.5 1.5 1.0 3.5 0.5 12.0 8.0]^*T*^

**Table 3:** Verification case II: critical data and design objectives.

Parameter	Description
Substrate	RO4003 (*ε*_*r*_ = 3.5, *h* = 0.51 mm)
Design parameters^$^	***x*** = [*L*_*s*_ *W*_*s*_ *l*_3*r*_ *w*_1_ *w*_2_ *w*_3_ *w*_4_ *w*_5_ *w*_*v*_]^*T*^
Other parameters^$^	*d*_*L*_ *= d*_*W*_ = 10 mm, *L =* 2*d*_*L*_ *+ L*_*s*_, *W =* 2*d*_*W*_ + 2*w*_1_ + (*W*_*s*_ – 2*w*_*f*_), *l*_1_ = *W*_*s*_/2, *l*_2_ = *l*_3_2^1/2^, *l*_3_ = *l*_*3r*_((*L*_*s*_ – *w*_3_)/2 – *w*_*4*_/2^1/2^), *l*_*v*1_ = *l*_3_/3, *l*_*v*3_ = *L*_*s*_/2 – *w*_3_/2 – *l*_3_ + *l*_*v*1_, *w*_*f*_ = 1.15 mm
EM model	CST Microwave Studio
Target operating frequencies [GHz]	*F*_1_ = [1.15 1.25] GHz *F*_2_ = [2.45 2.55] GHz
Design goals	Minimize matching |*S*_11_| and isolation |*S*_41_| at the two operating bands *F*_1_ and *F*_2_ Ensure power split ratio *K*_*P*_ = 0 dB at the operating frequencies *f*_0.1_ = 1.2 GHz, and *f*_0.2_ = 2.5 GHz
Parameter space *X*	***l*** = [0.4 0.6 3 9 0.6 0.4 0.1 0.6 4 0.6]^*T*^ ***u*** = [0.5 0.85 6.5 11 0.95 0.7 0.4 0.9 5 0.7]^*T*^

^$^Dimensions in mm, except relative one (with subscript *r*), which are unit-less.

**Table 4:** Verification case III: critical data and design objectives.

Parameter	Description
Substrate	AD250 (*ε*_*r*_ = 2.5, *h* = 0.81 mm)
Design parameters	***x*** = [*l*_1_ *l*_2_ *l*_3_ *l*_4_ *l*_5_ *s w*_2_]^*T*^
Other parameters	*w*_1_ = 2.2, *g* = 1
EM model	CST Microwave Studio
Target operating frequencies [GHz]	*F*_1_ = [2.95 3.05] GHz *F*_2_ = [4.75 4.85] GHz
Design goals	Minimize input matching |*S*_11_|, output matching |*S*_22_|, |*S*_33_|, and isolation |*S*_23_| at the two operating bands *F*_1_ and *F*_2_ Ensure equal power split ratio at the operating frequencies *f*_0.1_ = 3.0 GHz, and *f*_0.2_ = 4.8 GHz
Parameter space *X*	***l*** = [10.0 1.0 10.0 0.5 1.0 0.1 1.5]^*T*^ ***u*** = [40.0 20.0 40.0 15.0 6.0 1.5 8.0]^*T*^

### Test cases

Demonstration of the proposed procedure is realized based on three circuits:

- A compact branch-line coupler with microstrip cells (Circuit I)^53^;
- A dual-band branch-line coupler (Circuit II)^54^;
- A dual-band power divider (Circuit III)^55^.

The parameterized circuit architectures are illustrated in Fig. 4. Meanwhile, Tables 2 and 3, and Table 4 provide data concerning material and design parameters, design objectives, and search spaces for Circuit I, II, and III, respectively. The EM models are prepared in CST Microwave Studio and evaluated with the transient solver. For couplers, the objectives are to enhance the reflection characteristics (impedance matching) and port isolation over the prescribed frequency ranges (*F*_1_ for Circuit I, and *F*_1_/*F*_2_ for Circuit II) while ensuring equal power division at the band centers. For Circuit III, we aim to minimize the reflection co-efficient, enhance isolation at all pairs of ports (over the frequency bands F1 and F2), and ensure an equal power division ratio at the center frequencies. However, the second condition is fulfilled because of the circuit symmetry. Consequently, there is no need to explicitly handle it in the optimization process.

### Setup and results

**Fig. 5:**
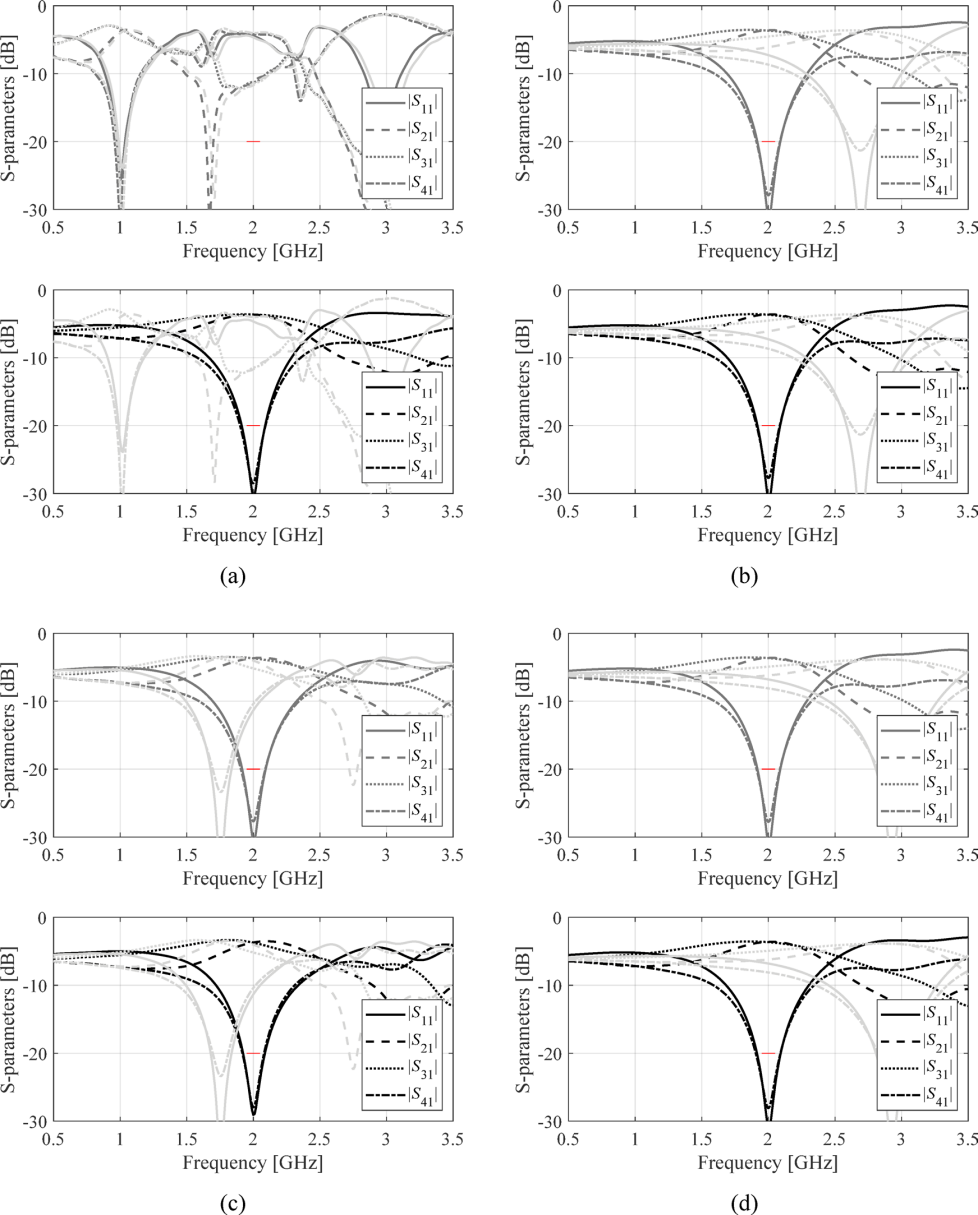
Circuit I: *S*-parameters the initial design (light grey), design found by conventional local search (dark grey), and design found using the proposed penalized optimization approach (black). Panels (**a**) through (**d**) show results obtained for starting points 1 through 4. The red lines mark the target operating band.

**Fig. 6:**
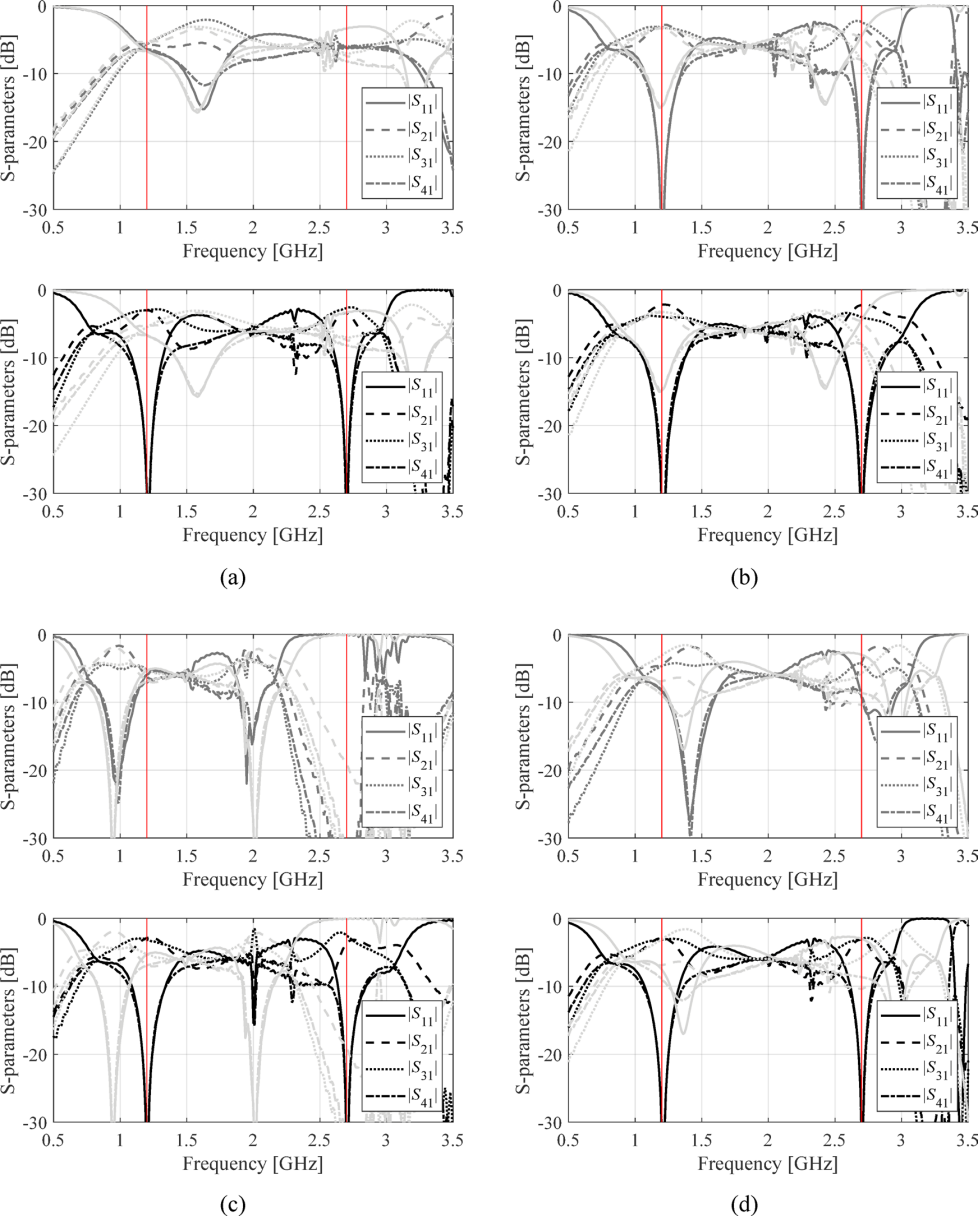
Circuit II: *S*-parameters the initial design (light grey), design found by conventional local search (dark grey), and design found using the proposed penalized optimization approach (black). Panels (**a**) through (**d**) show results obtained for starting points 1 through 4. Target operating frequencies denoted using vertical lines.

**Fig. 7:**
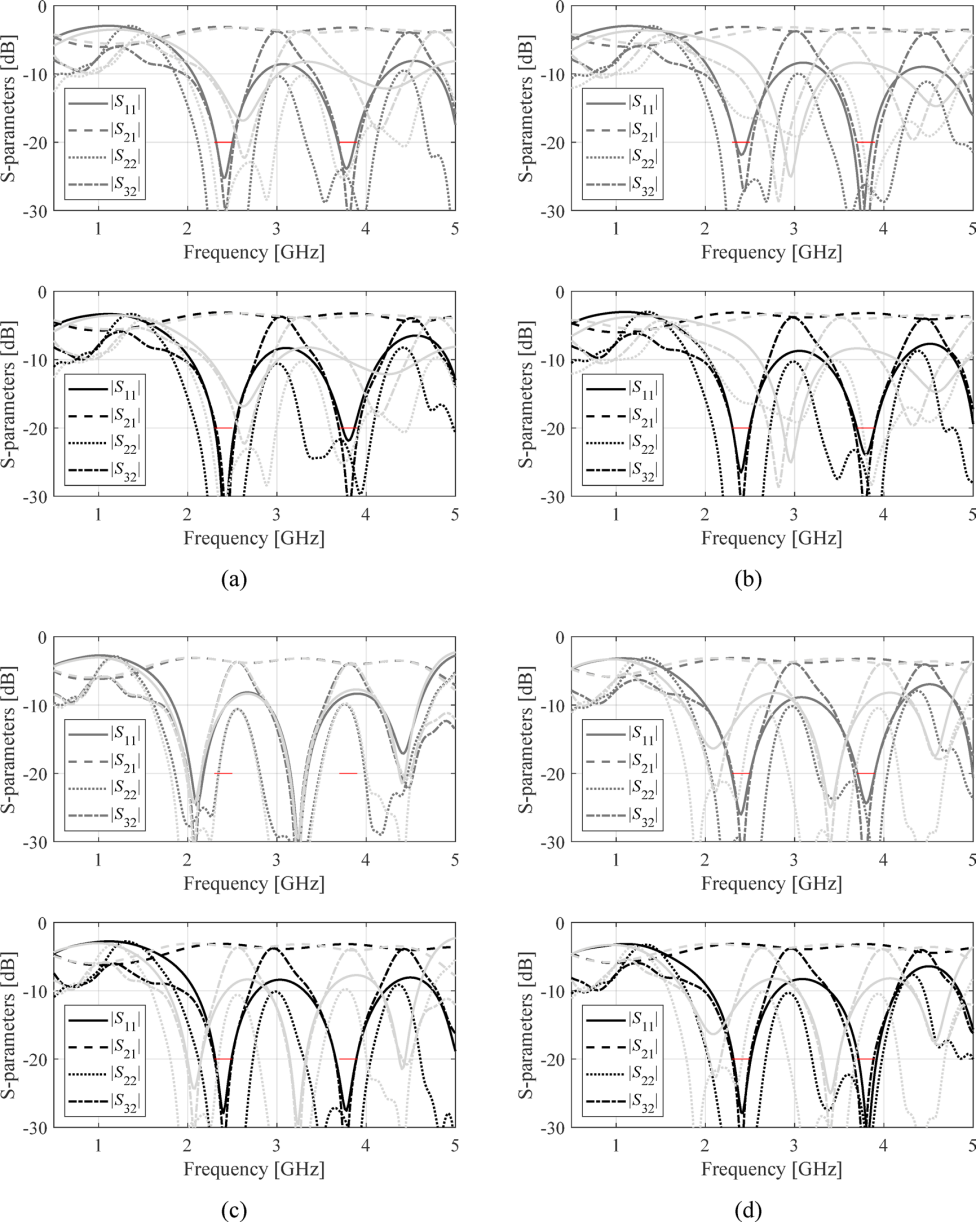
Circuit III: *S*-parameters the initial design (light grey), design found by conventional local search (dark grey), and design found using the proposed penalized optimization approach (black). Panels (**a**) through (**d**) show results obtained for starting points 1 through 4. Target operating bands denoted using the horizontal lines.

The structures in Fig. 4 were optimized assuming the conventional formulation (1), and the proposed penalized approach (2), (3). For each circuit, design objectives were set as described in Tables 2, 3 and 4, and the optimization was executed four times with different initial designs. The starting points were selected so that at least some of them are problematic for the conventional approach, as elaborated on in Sect. 2.2. The underlying search procedure is the TR routine outlined in Sect. 2.3. The scanning range has been set to *D*_*f*_ = 2 GHz. The results are encapsulated in Table 5. Figures 5, 6 and 7 illustrate the device outputs at the starting point and at the final designs, for Circuit I, II, and III.

**Table 5:** Optimization results.

Circuit			Starting point			
			1	2	3	4
I	Conventional formulation	Optimization successful?	No	Yes	Yes	Yes
		Final objective function value	–4.1 dB	–23.5 dB	–23.2 dB	–23.5 dB
	Penalized formulation (this work)	Optimization successful?	Yes	Yes	Yes	Yes
		Final objective function value	–23.8 dB	–23.5 dB	–22.9 dB	–23.8 dB
II	Conventional formulation	Optimization successful?	No	Yes	No	No
		Final objective function value	–6.2 dB	–29.9 dB	1.4 dB	–8.1 dB
	Penalized formulation (this work)	Optimization successful?	Yes	Yes	Yes	Yes
		Final objective function value	–28.6 dB	–29.1 dB	–32.0 dB	–30.7 dB
III	Conventional formulation	Optimization successful?	Yes	Yes	No	Yes
		Final objective function value	–18.8 dB	–17.8 dB	–3.9 dB	–19.8 dB
	Penalized formulation (this work)	Optimization successful?	Yes	Yes	Yes	Yes
		Final objective function value	–18.5 dB	–19.8 dB	–19.3 dB	–18.9 dB

For supplementary verification, our technique has been benchmarked against the particle swarm optimizer (PSO)^56^. PSO was set up in a conventional manner: swarm size *N* = 10, standard control parameters (*χ* = 0.73, *c*_1_ = *c*_2_ = 2.05); the number of iterations set to 100 (i.e., the computational budget is 1000 EM simulations). The algorithm was run ten times, leading to the following average objective function values (not that PSO is a global search algorithm; therefore, no initial design is required):

- Circuit I: − 15.3 dB,
- Circuit II: − 18.8 dB,
- Circuit III: − 12.1 dB.

These results are significantly worse than those obtained using the proposed methodology. For many runs, PSO could not identify designs meeting the assumed specs. Also, repeatability of results is poor (standard deviations across the set of ten algorithm runs is 3.1 dB, 5.2 dB, and 2.1 dB for Circuit I through III, respectively). These results further corroborate the benefits of the proposed methodology.

### Discussion

The results gathered in Table 5 demonstrate a consistent performance of the penalized design specification scanning approach introduced in Sect. 2. Some of the starting points have turned troublesome for the conventional approach, specifically those where the operating frequencies of the circuit were severely misaligned with the targets. Under those circumstances, the optimization process failed to produce satisfactory outcome. On the other hand, our methodology succeeded in all considered cases. Design specification scanning was capable to capture the actual location of the circuit center frequencies and—by combining information encoded in the conventional objective function *U* and the penalty term—enforce their relocation towards the target. This corroborates the improvement of the search process’ reliability, in particular, a reduction of a probability of failure under challenging scenarios, here, inferior initial designs.

The efficacy enhancement comes at a certain price, which is a slight increase in the search process cost. The expenses entailed by the suggested approach are about 27% higher than for the conventional approach; however, only for the optimization runs that were successful for the latter. In the case of failure, the cost is generally lower as the algorithm becomes quickly confined to the nearest local minimum. While the expenses entailed by our approach for the cases problematic for the conventional formulation are considerably higher. This is understandable as large-scale relocation of the center frequencies requires much larger budgets. Still these costs are significantly lower than those associated with any sort of global search procedures, especially nature-inspired algorithms.

A detailed breakdown of the CPU expenses follows. The mean costs of optimizing Circuit I are 92 and 89 EM analyses for conventional and penalized approaches, respectively. For Circuit I, the numbers are 250 and 158 EM analyses, whereas for Circuit III, we have 116 and 113 analyses, respectively. These figures indicate almost no cost increase for Circuits I and III. In contrast, it is noticeable for Circuit II, which is the most challenging case (recall that conventional formulation failed in three out of four runs). As mentioned earlier, the extra expenses are related to the necessity of relocating the operating frequencies of the circuit over a broad range of frequencies.

It should also be mentioned that the minimum cost of one iteration of the TR algorithm (penalized or not) is about *n* + 1 EM analyses (*n* simulations consumed by Jacobian estimation using finite differentiation). This cost may be slightly higher if the candidate design is not accepted, and the trust region size is reduced before launching the new iteration from the same starting point. The total optimization cost is then proportional to both the parameter space dimensionality and the number of algorithm iterations. The latter is generally higher for poor initial designs, which require more significant design relocation to identify the optimum.

Additional experiments were conducted for the first test case (Circuit I) to examine the effects of the scanning range *D*_*f*_ and shape coefficient *α*, on the optimization process performance. The results are summarized in Table 6. As shown, both *α* and *D*_*f*_ have only a minor impact on the optimization outcomes—differences are primarily due to numerical noise. An exception is observed for *α* = 2, where the penalized cost function becomes overly selective, resembling the conventional approach. In this case, the performance is slightly but noticeably diminished. Notwithstanding, it might also be possible to implement adaptive adjustment of these parameters, which will be the subject of future work. In practice, the scanning range *D*_*f*_ should be set relatively large to accommodate even relatively poor starting points––typically around one-third to half of the simulation range. As for the shape parameter *α*, its recommended value is 1. This value is a compromise between a highly selective penalty setup (*α* > 1) and a relaxed penalty arrangement (*α* < 1). The results in Table 6 corroborate this choice.

**Table 6:** Effects of control parameters *α* and *D*_*f*_.

Control parameters		Starting point			
*α*	*D*_*f*_	1	2	3	4
1	2 GHz	–23.8	–23.5	–22.9	–23.8
1	1 GHz	–23.1	–24.1	–23.1	–21.5
0.5	2 GHz	–23.5	–23.3	–21.8	–23.0
0.5	1 GHz	–22.2	–23.0	–23.3	–23.1
2	2 GHz	–19.8	–20.2	–22.0	–22.5
2	1 GHz	–20.1	–20.5	–20.1	–20.5

**Fig. 8:**
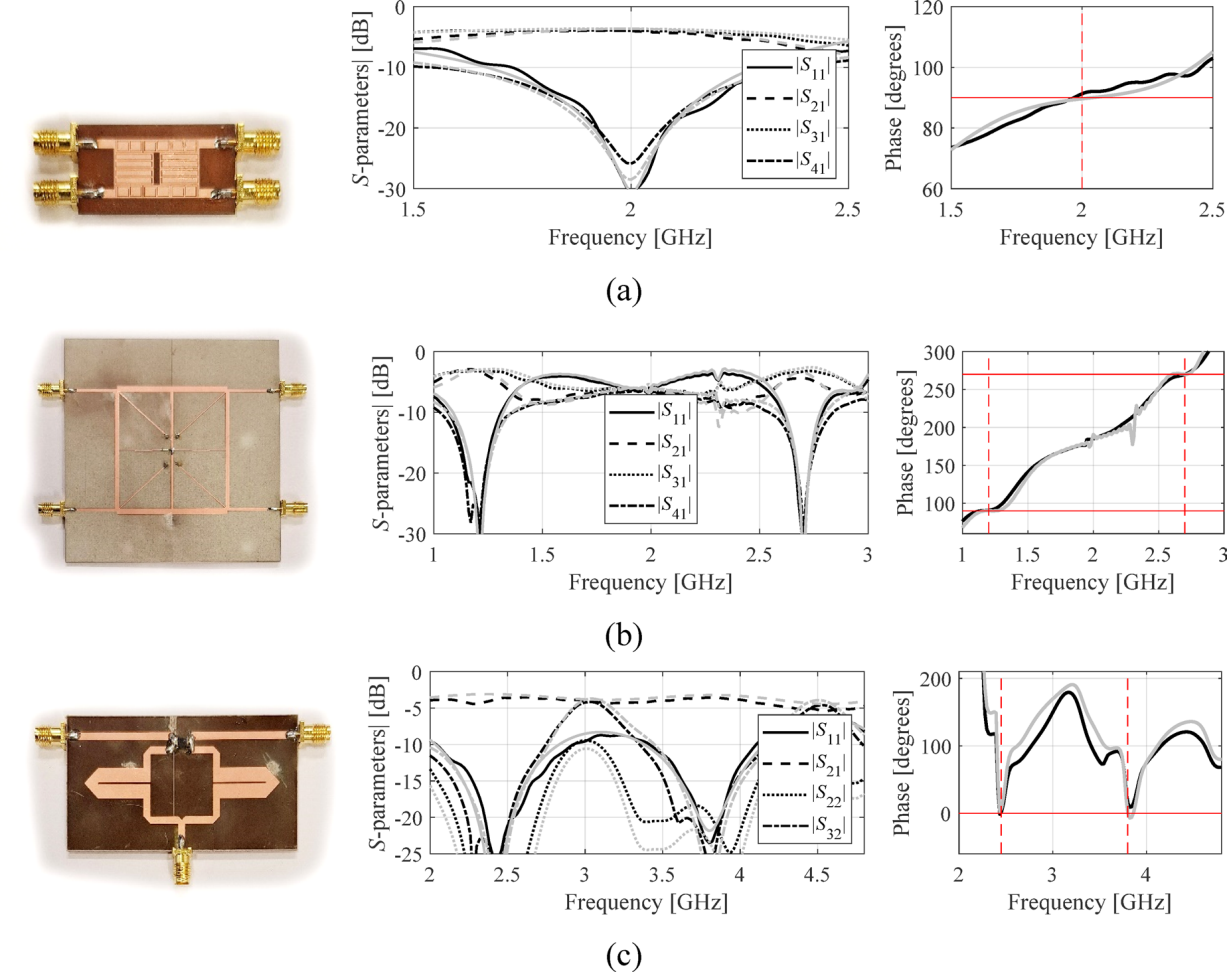
Experimental validation of optimized test circuits (all corresponding to the final designs found from the first initial points of Table 5). Dashed lines on phase responses mark the target operating frequencies, whereas horizontal lines mark target phase values: (**a**) Circuit I, (**b**) Circuit II, (**c**) Circuit III.

### Measurements

For additional verification, chosen designs of Circuits I, II, and III (all corresponding to the first initial point, cf. Table 5) were manufactured and measured. Figure 8 shows the circuit prototypes along with EM-simulated and experimentally validated frequency *S*-parameters. The alignment between the simulations and measurements is excellent. Only small misalignments can be observed, which are due to manufacturing tolerances and assembly imperfections.

## Conclusion

In this paper, we suggest a novel methodology for improving the reliability of parameter tuning of passive microwave structures. The presented technique is intended to be used with local optimization routines. Reliability improvement is understood in the sense of reducing the probability of failure under demanding scenarios, in particular, the unavailability of good starting points. Situations like this usually require defaulting to considerably more expensive global optimization methods (e.g., population-based metaheuristic algorithms), or repetitive optimization runs combined with manual re-adjustment of initial conditions. In contrast, the approach presented in this work reformulates the objective function by scanning the target operating bandwidths over the predefined frequency range, evaluating the objective function concerning the shifted bands, and adding a regularization term quantifying the discrepancy between the actual and intended center frequencies. This allows for capturing the circuit operating parameters and enforcing them towards the targets, thereby immunizing the optimization process against inferior initial designs.

The penalized objective function technique can be combined with any local search procedure. For verification, it has been incorporated into the trust-region algorithm and applied to several test cases that include three microstrip circuits and several initial designs (per circuit). The results obtained conclusively demonstrate reliability improvements enabled by the presented method. In particular, it allows the successful identification of the optimum design even for cases that are troublesome for conventional problem formulation, eventually leading to a failure. These benefits come with a certain increase in the optimization process cost, which is, however, minor. It is worth noting that our method is universal in the sense that it operates on the cost function, which is not directly related to the specific system under consideration. Therefore, it may be applied for antenna design purposes. Due to the nature of our technique, which is oriented toward facilitating identification of resonances or “sharp” minima, it is most suitable for narrow-band or multi-band systems.

## Data Availability

Data availability: The datasets generated during and/or analysed during the current study are available from the corresponding author on reasonable request. Contact person: anna.dabrowska@pg.edu.pl.
